# Role of Macrophage‐Related Genes GAS7 and ZEB2 in Acute Myocardial Infarction Pathogenesis

**DOI:** 10.1155/humu/5486574

**Published:** 2026-05-13

**Authors:** Zhenfang Liu, Jia Song, Lingling Li, Ziyin Fan, Genyuan Xie, Li Yang

**Affiliations:** ^1^ Department of Cardiovascular Medicine, The Affiliated Zhuzhou Hospital Xiangya Medical College, Central South University, Zhuzhou, China, csu.edu.cn

**Keywords:** acute myocardial infarction, diagnosis, high-dimensional weighted gene coexpression network analysis, macrophages, molecular docking, single-cell transcriptomics analysis

## Abstract

**Background:**

Acute myocardial infarction (AMI) involves complex immune responses and cellular interaction mechanisms. Although the pathogenesis of AMI is now preliminarily understood, there is still a lack of biomarkers that can accurately and rapidly diagnose its disease characteristics.

**Methods:**

This study analyzed single‐cell RNA sequencing (scRNA‐seq) and bulk RNA‐seq data related to AMI from the Gene Expression Omnibus (GEO) database. Data preprocessing and clustering were performed using Seurat, and cell–cell communication was analyzed using CellChat. Functional enrichment was performed using clusterProfiler. Key transcription factors were identified using SCENIC, and module‐specific genes associated with macrophages in AMI were identified using high‐dimensional weighted gene coexpression network analysis (hdWGCNA). These were combined with WGCNA to identify genes associated with AMI. Molecular docking was then used to predict potential targeted drugs for AMI. In addition, an oxygen–glucose deprivation (OGD)–induced AC16 cardiomyocyte model combined with quantitative real‐time polymerase chain reaction (qRT–PCR) was used to validate the expression patterns of the key regulators and biomarkers.

**Results:**

This study identified six cell types in AMI, including adipocytes, cardiomyocytes, endothelial cells, fibroblasts, macrophages, and smooth muscle cells. CellChat showed that cell–cell communication intensity was generally enhanced in AMI. hdWGCNA identified the M5 and M8 modules as significantly associated with macrophages. SCENIC analysis found that *FOS* and *ETV6* were important regulatory factors of macrophages. WGCNA screened the brown module as significantly associated with AMI, enriched in immune and inflammatory response pathways. The final integration of all analysis results identified *GAS7* and *ZEB2* as potential biomarkers for AMI, and receiver operating characteristic (ROC) curves validated the good diagnostic performance of the two genes for AMI. Furthermore, qRT–PCR in an OGD‐induced AC16 cardiomyocyte model confirmed the upregulation of *FOS*, *ETV6*, *GAS7*, and *ZEB2*, consistent with the transcriptomic findings. Molecular docking indicated that *GAS7* has a good binding affinity with sulforaphane.

**Conclusion:**

This study revealed the cellular communication characteristics in the pathogenesis of AMI and identified *GAS7* and *ZEB2* as potential biomarkers for AMI based on macrophage characteristics. It also elucidated their targeted therapeutic drugs, providing new insights into the mechanism and treatment of AMI.

## 1. Introduction

Acute myocardial infarction (AMI) is a life‐threatening condition caused by acute obstruction of the coronary arteries, leading to insufficient blood supply to the corresponding myocardial region and resulting in myocardial necrosis [1, 2]. Although current clinical diagnosis primarily relies on electrocardiogram changes and myocardial injury markers (such as troponin) testing, these methods still have significant limitations in terms of early diagnostic sensitivity and prognostic assessment, particularly in patients with atypical symptoms, where the diagnostic efficacy of existing biomarkers is often insufficient [3–5]. Additionally, traditional biomarkers struggle to fully reflect the complex pathophysiological processes of AMI, including key components such as inflammatory responses, oxidative stress, and myocardial remodeling [6, 7]. Therefore, identifying novel biomarkers with higher sensitivity and specificity holds significant clinical importance for achieving early, precise diagnosis and personalized treatment of AMI.

Macrophages are key effector cells of the innate immune system, and resident cardiac macrophages are responsible for the efficient clearance and degradation of apoptotic myocardial cells, playing a crucial role in inflammatory regulation and tissue repair following AMI [8]. Studies have shown that tissue‐resident cardiac macrophages can be divided into the CCR2‐subpopulation derived from embryos and the CCR2+ subpopulation derived from the adult hematopoietic lineage [9]. Among these, the CCR2‐subpopulation primarily promotes coronary artery development, cardiac regeneration, and facilitates signal transmission within the atria and ventricles [10]. CCR2+ subpopulation macrophages primarily originate from monocytes. Related studies have revealed that these macrophages can promote monocyte recruitment, release of monocyte chemotactic protein (MCP), and cell mobilization through a MYD88‐dependent mechanism [11]. Studies have shown that macrophages rapidly infiltrate the infarct area after myocardial ischemia and participate in different stages of pathophysiological processes through phenotypic polarization. Among them, proinflammatory M1 macrophages exacerbate early myocardial damage, whereas anti‐inflammatory M2 macrophages promote late tissue repair, and the dynamic balance between the two directly affects the process of myocardial remodeling [12]. From a regulatory mechanism perspective, macrophages stimulated by proinflammatory signals such as lipopolysaccharides and interferons typically exhibit enhanced phagocytic activity and antigen presentation functions. They can induce a proinflammatory microenvironment through proinflammatory cytokines like IL‐12 and IL‐23, as well as chemokines such as CXCL9 and CXCL10, thereby affecting AMI repair [13]. In contrast, M2 macrophages selectively produce anti‐inflammatory cytokines such as IL‐10, which stimulate fibroblast‐mediated extracellular matrix production and cell proliferation, thereby promoting myocardial tissue remodeling and repair [14]. Therefore, using macrophage‐related characteristics as a starting point not only reveals the key molecular mechanisms of AMI but also provides a unique perspective for developing molecular targets with both diagnostic value and therapeutic potential.

This study analyzed the single‐cell atlas of AMI and clarified the pivotal role of macrophages in AMI pathogenesis. By integrating weighted gene coexpression network analysis (WGCNA) with other computational approaches, we identified macrophage‐ and AMI‐related key genes as potential diagnostic biomarkers, and their dysregulated expression was further validated in an oxygen–glucose deprivation (OGD)–induced AC16 cardiomyocyte model. Subsequent molecular docking suggested candidate therapeutic agents targeting these biomarkers, providing a preliminary basis and important reference for the development of new diagnostic and therapeutic strategies for AMI.

## 2. Materials and Methods

### 2.1. Data Acquisition

The single‐cell multiomics dataset GSE270788 and the bulk microarray dataset GSE66360 were obtained from the Gene Expression Omnibus (GEO) database (http://www.ncbi.nlm.nih.gov/geo). We obtained cardiac tissue samples from five AMI cases and seven healthy controls in the GSE270788 dataset. Additionally, we obtained samples from the GSE66360 dataset, which includes 50 healthy controls and 49 MI cases. Both datasets are publicly available, and no specific permission was required for their use in this study.

### 2.2. Single‐Cell Data Analysis

For the GSE270788 dataset, we used the Seurat package [15] to read the scRNA‐seq data, retaining cells with a mitochondrial gene proportion < 7.5% and a gene count between 200 and 3000, resulting in a total of 28,619 cells. We then standardized the data using the ScaleData function, performed principal component analysis (PCA) dimensionality reduction, and used the harmony package to remove batch effects between different samples. After dimensionality reduction using the first 20 principal components via uniform manifold approximation and projection (UMAP), we clustered the cell subpopulations using the FindNeighbors and FindClusters functions (resolution = 0.2). Cell types were determined based on the expression of known marker genes from the CellMarker database.

### 2.3. Cell Communication

This study used the CellChat package [16] to explore ligand–receptor pairs in cell subpopulation interactions, using bubble plots to display the likelihood and significance of ligand–receptor interactions between cell subpopulations.

### 2.4. Enrichment Analysis

This study used the FindMarkers function to identify differentially expressed genes between different cell subpopulations and used the gseGO function of the clusterProfiler package [17, 18] to perform biological process enrichment analysis on gene sets sorted by logFC.

### 2.5. Transcription Factor Analysis

In this study, the human RcisTarget database was downloaded from the website (https://resources.aertslab.org/cistarget/) for the construction of the transcription factor regulatory network. The network was constructed using the R package SCENIC [19]. A regulon is a set of target genes regulated by the same TF, and the AUCell algorithm [19] was used to evaluate transcription factor activity.

### 2.6. High‐Dimensional Weighted Gene Coexpression Network Analysis (hdWGCNA)

In this study, AMI sample cell clusters were selected from scRNA data, and only genes expressed in ≥ 5% of cells were retained to create an initial WGCNA object. Subsequently, the KNN algorithm with *k* = 25 was used to aggregate cells, integrating up to 20 neighboring cells into a single metacell to reduce the sparsity of the single‐cell expression matrix. The optimal soft threshold power was automatically selected using pickSoftThreshold(), and a weighted coexpression network was constructed based on this. Module feature relationship analysis identified modules significantly associated with macrophages, and key genes in important modules were identified based on their intramodule connectivity. The Top 15 genes were considered to be module feature genes associated with macrophages.

### 2.7. WGCNA Identifies AMI‐Related Genes

The GSE66360 dataset was used in WGCNA [20] to identify gene modules associated with AMI. First, the samples were clustered to identify coexpression modules. The study demonstrated that the coexpression network conforms to a scale‐free network, where the logarithm of the degree *k* of a node (log(k)) is negatively correlated with the logarithm of the probability of the node′s occurrence (log(P(k))), with a correlation coefficient exceeding 0.85. To ensure the network is scale‐free, the soft threshold *β* is determined using the pickSoftThreshold() function. Next, the expression matrix is converted into an adjacency matrix, which is then converted into a topological matrix. Based on topological overlap matrix (TOM), genes are clustered using average‐linkage hierarchical clustering.

### 2.8. Molecular Docking

In this study, the intersection of SCENIC analysis results, macrophage‐related module characteristic genes, and AMI‐related genes was obtained to identify potential biomarkers for AMI in this study. The receiver operating characteristic (ROC) curve and area under the curve (AUC) analysis were performed using the R package pROC to evaluate the diagnostic performance of each biomarker for the disease. Three‐dimensional structures of drug molecules were downloaded from the PubChem website (https://pubchem.ncbi.nlm.nih.gov/) as ligands, and the three‐dimensional structures of targeted drugs were minimized using the ChemBioOffice software. Receptor protein crystal structures were obtained from the Uniprot database (https://www.uniprot.org/), and 3D structure files of target proteins were downloaded from the PBD database (https://www.rcsb.org/). Water molecules and ligands were removed using Pymol software [21], and molecular docking was performed using AutoDockTools software. The screening criteria were as folows: binding energy < −5 kcal/mol.

### 2.9. Cell Culture and OGD Treatment

Human cardiomyocytes AC16 were used in this study. Cells were maintained in Dulbecco′s modified Eagle′s medium (DMEM) with high glucose supplemented with 10% fetal bovine serum (FBS) and 1% penicillin–streptomycin under standard culture conditions (37°C, 5% CO_2_, saturated humidity) in a humidified incubator. Cells were passaged at 70%–80% confluence using Trypsin–EDTA and were routinely tested to be mycoplasma‐free.

For establishment of the OGD–reoxygenation model, AC16 cells were seeded in appropriate culture plates and grown to approximately 70%–80% confluence. After the removal of the complete medium, cells were gently rinsed two to three times with glucose‐free phosphate‐buffered saline (PBS) to thoroughly eliminate residual high‐glucose medium. Prewarmed glucose‐free DMEM was then added, and the cells were transferred to a hypoxic incubator (37°C, 1% O_2_, 5% CO_2_, 94% N_2_) for 2–6 h to induce OGD [22, 23]. At the end of hypoxic incubation, the glucose‐free medium was discarded and replaced with high‐glucose DMEM containing 10% FBS, and the cells were returned to a normoxic incubator (37°C, 5% CO_2_) for 24 h to mimic reperfusion injury. Control cells were maintained in high‐glucose DMEM with 10% FBS under standard normoxic conditions throughout the same period.

### 2.10. Quantitative Real‐Time PCR (qRT–PCR)

Total RNA was extracted from cultured cells using TRIzol reagent (15596026CN; Invitrogen, Carlsbad, California, United States) in accordance with the manufacturer′s protocol. RNA yield and quality were assessed with a NanoDrop spectrophotometer (Thermo Fisher Scientific, United States). Complementary DNA (cDNA) was generated from total RNA using a reverse transcription kit (D7178S, Beyotime, Shanghai, China). Quantitative PCR was subsequently performed on an Applied Biosystems real‐time PCR platform (Applied Biosystems, Foster City, California, United States) using SYBR Green qPCR Mix (D7260, Beyotime, China).

The amplification conditions were as follows: an initial denaturation at 95°C for 10 min, followed by 30 cycles of 95°C for 15 s, 60°C for 30 s and 72°C for 1 min, and a final extension at 72°Cfor 5 min. Relative mRNA expression levels were calculated using the 2^−*Δ* *Δ*Ct^ method, with *GAPDH* serving as the endogenous control. Primer sequences are listed in Table 1. All samples were run in technical triplicates, and no‐template controls were included in each assay to monitor specificity and exclude contamination.

**Table 1:** The primers utilized in this study.

Gene	Accession no.	Primers (5 ^′^–3 ^′^)	
		Forward	Reverse
*FOS*	NM_005252	GCCTCTCTTACTACCACTCACC	AGATGGCAGTGACCGTGGGAAT
*ETV6*	NM_001987	GAATGGCAAAGCTCTCCTGCTG	TCCGAGGTTTCCTCTGCTTCAG
*GAS7*	NM_201433	CTCTCAGAACTCCTTGGCTTCAC	GTTCTCACGGAAGTTCATCAGGG
*ZEB2*	NM_014795	AATGCACAGAGTGTGGCAAGGC	CTGCTGATGTGCGAACTGTAGG
*GAPDH*	NM_002046	GTCTCCTCTGACTTCAACAGCG	ACCACCCTGTTGCTGTAGCCAA

### 2.11. Statistical Analysis

All statistical analyses were conducted using R software (Version 4.2.0). Differences in continuous variables between two groups were assessed with the Wilcoxon rank‐sum test. All cellular experiments were performed in at least three independent biological replicates. qRT–PCR data were analyzed using two‐way analysis of variance (ANOVA) to evaluate the effects of treatment (control vs. OGD) and gene expression levels. When a significant interaction or main effect was detected, Bonferroni′s multiple comparisons test was applied to adjust for multiple testing. Data are presented as mean ± standard deviation (SD). A two‐sided *p* value < 0.05 was considered to indicate statistical significance.

## 3. Results

### 3.1. AMI Single‐Cell Atlas

**Figure 1:**
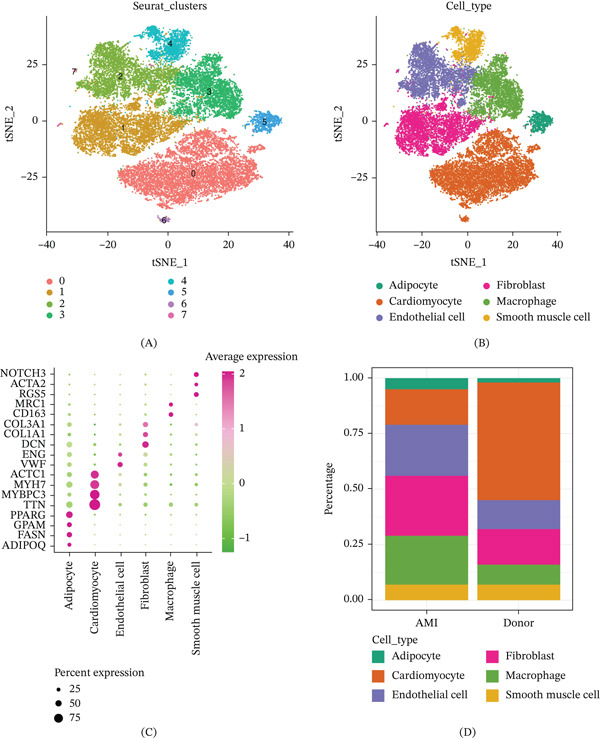
Single‐cell clustering of AMI. (A–B) Construction of single‐cell maps of AMI and control samples. (C) Bubble chart showing the expression levels of marker genes for each cell type. (D) Differences in cell proportions between AMI and control samples.

After cell filtering, standardization, dimensionality reduction, and clustering, we identified a total of eight clusters. Through cell annotation using the cellmarkar2.0 website, we ultimately obtained six cell types: adipocyte, cardiomyocyte, endothelial cell, fibroblast, macrophage, and smooth muscle cell (Figure 1A–C). By comparing the differences in cell proportions between AMI and donor samples, we found that compared with healthy controls, AMI had a lower proportion of cardiomyocytes and higher proportions of macrophages, fibroblasts, and adipocytes (Figure 1D).

### 3.2. Analysis of Cell–Cell Communication in AMI Cell Subpopulations

**Figure 2:**
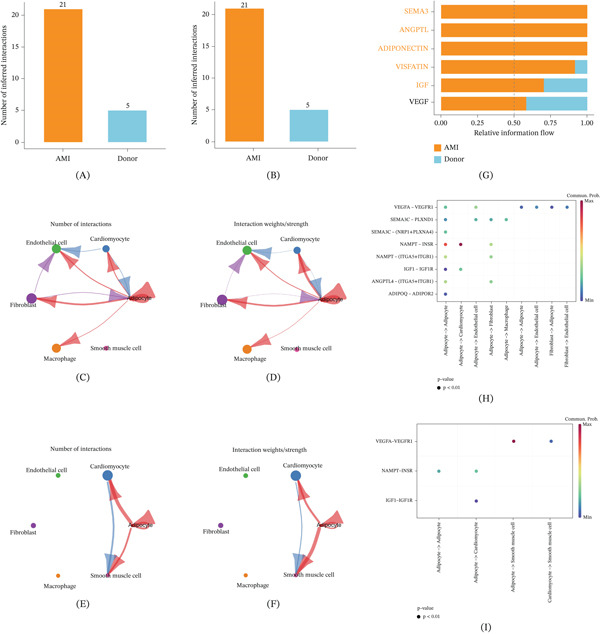
Analysis of communication between cells in AMI. (A–B) Comparison of the number and intensity of cell communication events between AMI and the control group. (C–D) Network diagram of the number and intensity of intercellular communication in the AMI group, with red arrows indicating strong communication and thicker arrows indicating higher weight. (E–F) Communication network diagram between cell types in the control group. (G) Comparison of signaling pathways with significant changes in relative information flow. (H–I) Bubble plot of ligand–receptor pairs in AMI and control samples, with dot size indicating *p* value and color indicating significance.

To further elucidate the cell–cell communication relationships in AMI, we analyzed single‐cell transcriptomic data using CellChat and systematically compared it with a healthy control group. The results showed that the number and intensity of cell–cell communication in the AMI group were significantly higher than those in the control group (Figure 2A–B). Further analysis at the cellular type level revealed that communication intensity between fibroblasts, cardiomyocytes, and macrophages was significantly enhanced in the AMI group, particularly in terms of signal flow toward endothelial cells, whereas such communication was weaker in the healthy control group (Figure 2C–F). At the signaling pathway level, the relative information flow in pathways related to SEMA3, ANGPTL, and ADIPONECTIN was significantly higher in the AMI group than in the healthy control group (Figure 2G). Key ligand–receptor pair analysis further revealed highly active intercellular communication events in AMI, including enhanced axes such as SEMA3C − PLXND1, SEMA3C − (NRP1 + PLXNA4), and NAMPT − (ITGA5 + ITGB1) (Figure 2H–I).

### 3.3. Functional Enrichment of Cell Subpopulations in AMI

**Figure 3:**
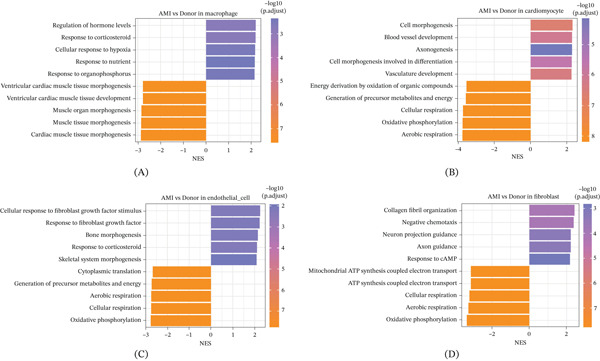
GSEA analysis results: (A) GSEA analysis of macrophages, (B) cardiomyocytes, (C) endothelial cells, and (D) fibroblasts in AMI.

We performed GSEA on macrophages, cardiomyocytes, endothelial cells, and fibroblasts in AMI. In macrophages, multiple pathways related to stress and metabolism were significantly activated in the AMI group, including response to nutrients, response to corticosteroids, and regulation of hormone levels. Some developmental pathways related to cardiac muscle morphogenesis, such as cardiac muscle tissue morphogenesis and ventricular cardiac muscle tissue development, were suppressed in AMI (Figure 3A). In cardiomyocytes, mitochondrial metabolic pathways such as aerobic respiration, oxidative phosphorylation, and cellular respiration were inhibited in AMI, whereas developmental pathways such as blood vessel development and cell morphogenesis were activated in AMI (Figure 3B). In endothelial cells, energy metabolism‐related pathways were inhibited, whereas the response to fibroblast growth factor was activated in AMI (Figure 3C). In fibroblasts, pathways related to mitochondrial metabolism and respiration were inhibited in AMI, whereas pathways such as response to cAMP, axon guidance, and collagen fibril organization were activated in AMI (Figure 3D).

### 3.4. Gene Screening of Modules Related to Macrophages in AMI

**Figure 4:**
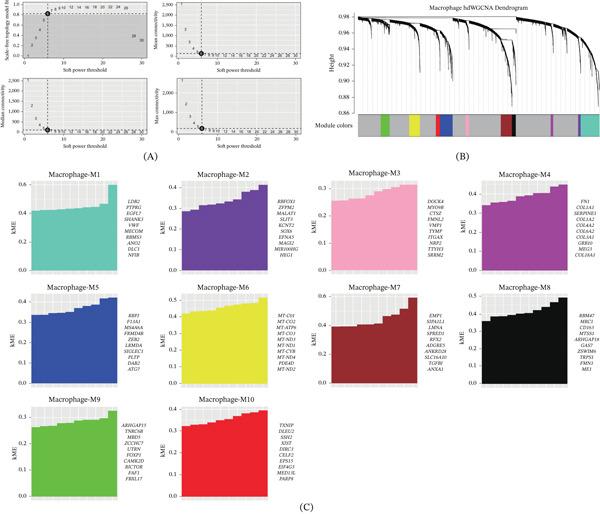
hdWGCNA screening of macrophage‐related modules in AMI. (A) Soft threshold screening, with the optimal soft threshold in the figure being 6. (B) Coexpression network tree, with the upper part representing the gene tree, where each branch corresponds to a gene, and the lower part representing the modules associated with each gene. (C) Module partitioning, with the vertical axis representing kME values, indicating the connectivity of each gene based on feature genes and the right side showing the hub genes of each module.

This study focused on macrophage subpopulations in AMI as the analysis subject. The pickSoftThreshold() function was used to automatically screen for the optimal soft threshold power *β* = 6, and a weighted gene coexpression network was constructed based on this (Figure 4A,B). By calculating the module feature vector (ME) and gene internal connectivity, the data were ultimately divided into 10 coexpression modules, and the hub genes of each module were identified (Figure 4C).

**Figure 5:**
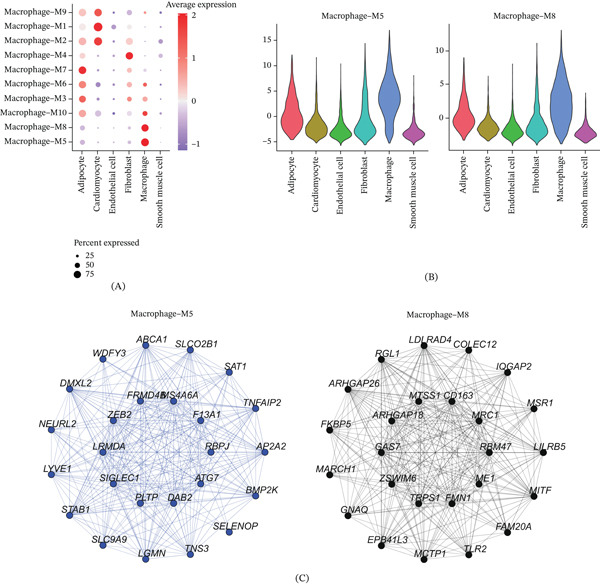
Expression of genes within modules in different cells. (A) Expression levels of genes within modules in different cells, with red representing high expression, purple representing low expression, and circle size representing cell proportion. (B) Violin plots of module feature value expression distributions in each cell for coexpression Modules M5 and M8. (C) Coexpression network of hub genes in coexpression Modules M5 and M8.

Subsequently, we calculated the expression levels of genes within each module in different cell types. We found that genes in Modules M5 and M8 exhibited higher expression proportions and levels in macrophages (Figure 5A,B). Therefore, we selected these two modules as key modules and extracted the hub gene networks within them (Figure 5C).

### 3.5. Identification of Key Transcription Factors in Macrophages

**Figure 6:**
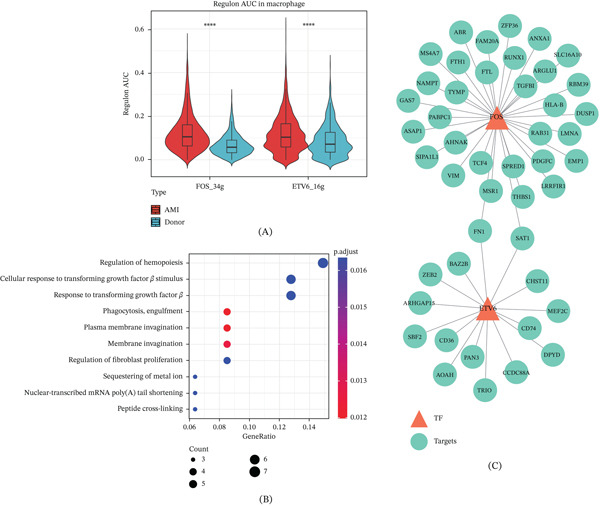
Transcription factor identification in macrophages. (A) Distribution of key regulatory factor activity in AMI and donor samples. (B) GO functional enrichment analysis of the regulon target gene set. (C) Transcription factor–target gene regulatory network diagram, with orange triangles representing transcription factors and green circles representing target genes.

This study identified two highly significant regulatory factors in macrophages: *FOS* and *ETV6*, both of which showed significantly higher regulatory activity in AMI patients than in the control group (Figure 6A). Further enrichment analysis of the target gene sets for the two regulons revealed that these downstream genes were significantly enriched in processes such as regulation of hemopoiesis, cellular response to transforming growth factor, membrane invagination, and regulation of fibroblast proliferation (Figure 6B). Finally, this study constructed a regulatory network diagram linking the key regulatory factors to their target genes (Figure 6C).

### 3.6. WGCNA Identification of Genes Associated With AMI

**Figure 7:**
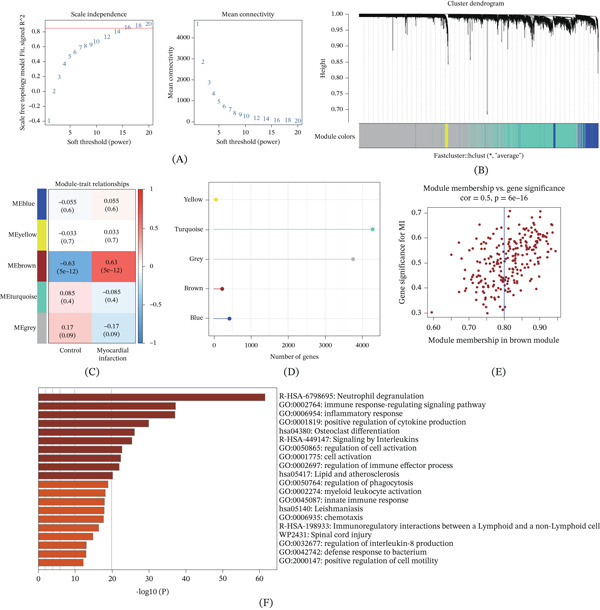
WGCNA analysis results of AMI‐related genes. (A) Soft threshold selection was used to determine the scale‐free nature of the network. (B) The gene clustering dendrogram shows the distribution of different modules. (C) Heat map showing the correlation between different gene modules and AMI and the control group. (D) Number of genes in each module. (E) Scatter plot showing the correlation between gene module membership and gene significance in the brown module. (F) Functional enrichment analysis of genes in the brown module.

In this study, WGCNA was performed on GSE66360 to screen for AMI‐associated genes. Appropriate parameters were determined through soft threshold screening, with the scale‐free topological fitting index reaching stability at a soft threshold of 16 (Figure 7A). Based on this, hierarchical clustering was performed on the genes, and a clustering dendrogram was generated (Figure 7B). Among these, the brown module showed the strongest positive correlation with AMI (Figure 7C). The number of genes in each module is shown in Figure 7D. According to the screening criteria (MM > 0.8 and GS > 0.2), 133 genes with high connectivity were identified as hub genes (Figure 7E). Gene enrichment analysis of the brown module revealed significant enrichment in immune regulation–related pathways such as neutrophil degranulation, immune response–regulating signaling pathways, and inflammatory response (Figure 7F).

### 3.7. Identification of Key Biomarkers Associated With AMI and Molecular Docking

**Figure 8:**
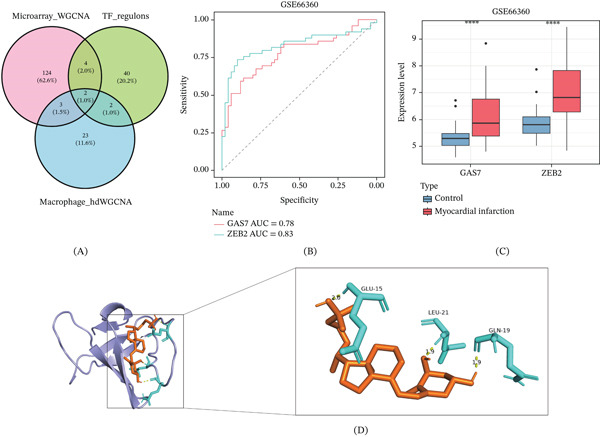
Molecular docking of biomarkers. (A) Venn diagram showing the intersection of key candidate genes screened by three methods: hdWGCNA, TF regulatory network, and macrophage‐specific WGCNA. (B) ROC curves for key genes. (C) Expression of key genes in GSE66360. (D) Molecular docking results of *GAS7* and sulforaphane. Blue molecules represent receptor proteins, orange molecules represent drug small molecules, and cyan represents amino acids. Hydrogen bonds between receptor proteins and drug small molecules are indicated by yellow dashed lines, with the numbers above representing hydrogen bond lengths.

In this study, genes screened by WGCNA, SCENIC analysis, and hdWGCNA were intersected to obtain *GAS7* and *ZEB2* as potential biomarkers for AMI (Figure 8A). In the GSE66360 dataset, ROC analysis validated the good diagnostic performance of the two genes in AMI, with AUC values of 0.78 and 0.83, respectively (Figure 8B). *GAS7* and *ZEB2* were both significantly overexpressed in the AMI group (Figure 8C).

In the subsequent analysis, we predicted the potential drug–protein interaction networks for *GAS7* and *ZEB2*, identified sulforaphane as a target compound, and performed molecular docking analysis using the crystal structures 2lx7 of *GAS7* and 2da7 of *ZEB2* (Table 2). The results showed that the binding energy between *GAS7* and sulforaphane was −7.41 kcal/mol, indicating good binding stability (Figure 8D).

**Table 2:** Molecular docking parameters.

Compound CID	Molecula_name	Gene_name	PDB_ID	Energy (kcal/mol)
5350	Sulforafan	GAS7	2lx7	−7.41
5350	Sulforafan	ZEB2	2da7	−4.71

### 3.8. Validation of *FOS*, *ETV6*, *GAS7*, and *ZEB2* Expression in an OGD‐Induced Injury Model

**Figure 9:**
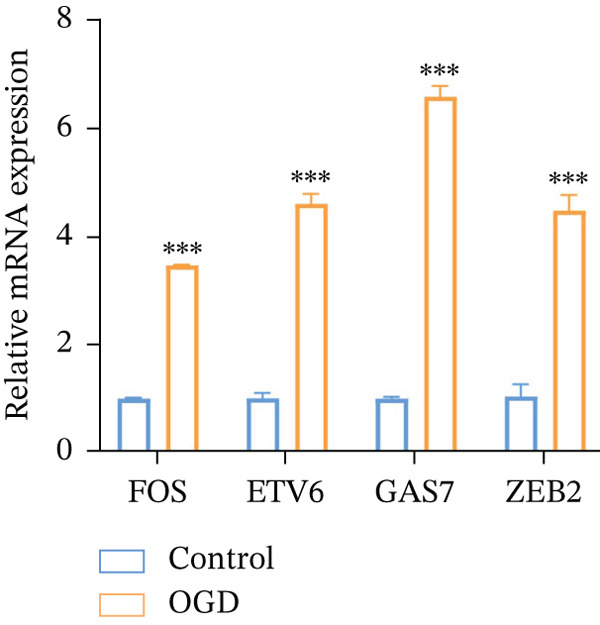
Validation of key genes in an OGD‐induced injury model. Relative mRNA expression levels of *FOS*, *ETV6*, *GAS7*, and *ZEB2* in control and OGD‐treated cells measured by qRT–PCR (*n* = 3). Data are presented as mean ± SD.  ^∗∗∗^ *p* < 0.001 versus control.

To experimentally validate the expression patterns of the key regulators and biomarkers identified by integrative bioinformatics analyses, we established an OGD model in vitro. qRT–PCR showed that the mRNA levels of *FOS*, *ETV6*, *GAS7*, and *ZEB2* were all significantly upregulated in OGD‐treated AC16 compared with the control group (Figure 9). These results are consistent with the transcriptomic findings and further support the involvement of these genes in ischemic myocardial injury.

## 4. Discussion

The pathogenesis of AMI is complex, involving inflammatory responses, immune cell infiltrations, and interactions between various cell types [24–26]. In recent years, research has increasingly recognized the important role of the immune system, particularly innate immune cells, such as macrophages, in the treatment of AMI. Macrophages not only participate in the clearance and repair of necrotic myocardium but also influence tissue remodeling and functional recovery in the late stages of myocardial injury through cytokine secretion and regulation of cellular communication [27–29]. However, the molecular mechanisms underlying AMI remain unclear, and there is a lack of stable and effective diagnostic biomarkers and treatment strategies [30]. Here, we integrate single‐cell transcriptomic data with conventional transcriptomic data for the first time to systematically analyze the cellular communication networks and transcriptional regulatory mechanisms of AMI from a macrophage perspective. By innovatively combining multilevel bioinformatics methods such as hdWGCNA, SCENIC, and WGCNA, we have identified *GAS7* and *ZEB2* as novel diagnostic biomarkers for AMI closely associated with macrophages. These findings provide new candidate biomarkers for the early diagnosis of AMI and are expected to advance the development of precision diagnostics and personalized treatment strategies for AMI.

This study identified two highly significant regulatory factors in macrophages: *FOS* and *ETV6*, both of which exhibited significantly higher regulatory activity in AMI patients compared with the control group. Upregulation of *FOS* plays a crucial role in myocardial lesions and may be involved in the pathogenesis of AMI, as demonstrated by a related study where metoprolol inhibition of *FOS* expression in rats showed positive therapeutic effects on AMI rats [31]. Additionally, *FOS* in macrophages is associated with tissue repair and inflammatory progression following myocardial infarction. For example, the synergistic effect of Lgr4 in inflammatory phenotype macrophages on AP‐1 activation is achieved through the transactivation of *FOS*, thereby influencing tissue repair and exacerbating inflammatory progression [32]. *ETV6* encodes a transcription repressor factor of the E26 transformation‐specific family. Recent reports indicate that variants in *ETV6* are responsible for hereditary thrombocytopenia and hematological malignancies, as *ETV6* functions as a cytoskeletal regulatory gene in platelet production, leading to alterations in platelet size, shape, and function, as well as changes in circulating progenitor cell levels, which have adverse effects on cardiovascular disease [33]. *ETV6* in macrophages is associated with NF‐*κ*B pathway activation and the expression of IL‐1*β*, IL‐6, and TNF‐*α*, playing a role in the atherosclerosis process by promoting plaque formation, local inflammation, and thrombosis [34]. As such, both regulatory factors are closely associated with macrophage phenotype regulation, consistent with the findings of this study, which indicate that the target genes of these two regulatory factors are significantly enriched in pathways related to cellular phenotype regulation, such as the cellular response to transforming growth factor, thereby influencing AMI.

This study further screened candidate genes using WGCNA and ultimately identified *GAS7* and *ZEB2* as potential biomarkers in AMI. Notably, the upregulation of *FOS*, *ETV6*, *GAS7*, and *ZEB2* was further validated by qRT–PCR in an OGD‐induced AC16 cardiomyocyte model, which mirrored the dysregulation patterns identified in public AMI datasets and provided experimental support for our integrative bioinformatics findings. A study found that *GAS7* was significantly upregulated in M1 polarization of macrophages, accompanied by activation of the NF‐*κ*B pathway in cells, whereas knockdown of *GAS7* reduced M1 polarization and expression of M1 marker genes [35]. This indicates that *GAS7* is closely associated with the proinflammatory phenotype of macrophages, and proinflammatory macrophages have adverse effects on both early myocardial injury and subsequent tissue repair in AMI [36]. Myocardial cells stimulate angiogenesis in a *ZEB2*‐dependent manner following ischemic injury. Specifically, myocardial cells in the infarcted heart induce the expression of *TMSB4* and *PTMA* by expressing *ZEB2*, thereby enhancing endothelial cell migration to stimulate angiogenesis and prevent heart failure [37]. Thus, the genes screened based on macrophage characteristics in this study are associated with cardiovascular disease regulation or cardiomyocyte phenotype and may have potential diagnostic value for AMI.

This study further predicted the potential drug–protein interaction network of *GAS7* and *ZEB2*, identifying sulforaphane as a target compound, with *GAS7* exhibiting good binding stability with sulforaphane. Sulforaphane is the most common antioxidant component in cruciferous plants [38, 39]. Previous studies have shown that this compound can delay the progression of inflammation‐induced liver fibrosis by regulating intracellular oxidative stress levels, involving the JAK/STAT3 pathway [40]. In the regulation of cardiovascular‐related diseases, sulforaphane inhibits myocardial cell apoptosis and oxidative stress in myocardial cells, thereby alleviating myocardial damage [41]. An animal experiment validated sulforaphane′s excellent cardiac protective effects, demonstrating that long‐term low‐dose sulforaphane treatment of fibroblasts enhances the release of F‐EXO exosomes targeting cardiomyocytes, thereby effectively preventing the onset of heart failure. This opens a new avenue for the large‐scale production of cardiac protective exosomes using fibroblasts for clinical applications [42]. This also validates the efficacy of sulforaphane as a potential therapeutic agent for AMI.

This study also has certain limitations. First, the study data were primarily derived from public databases, with a relatively limited sample size and a lack of external independent validation cohorts. In the future, it will be necessary to collect clinical samples from AMI patients across multiple centers and with a large sample size to validate the robustness and universality of *GAS7* and *ZEB2* as diagnostic biomarkers through prospective cohort studies. Second, the specific molecular mechanisms by which *GAS7* and *ZEB2* regulate macrophage function and AMI progression have been inferred solely through bioinformatics predictions, with a lack of direct evidence at the tissue level or in animal models. Future studies should establish macrophage‐specific *GAS7*/*ZEB2* knockout mice or utilize lentivirus‐mediated gene overexpression/silencing techniques, combined with AMI animal models and experimental methods such as immunohistochemistry and Western blotting, to elucidate the signaling pathways involved in macrophage polarization, cytokine secretion, and myocardial repair. Third, molecular docking suggests that sulforaphane binds well to GAS7; however, its therapeutic efficacy as a targeted treatment has not yet been validated at the cellular or animal level. Therefore, future studies should validate the direct binding between the two using surface plasmon resonance (SPR) or cell‐based thermal shift analysis (CETSA), and evaluate the effects of sulforaphane administration on *GAS7* expression, macrophage phenotype, and cardiac function parameters in OGD‐induced cardiomyocytes or AMI animal models to clarify its clinical translation potential as a drug candidate. Through these targeted studies, it is anticipated that the preliminary findings of this research can be advanced toward clinical diagnostic and therapeutic applications.

## 5. Conclusion

In summary, this study utilized single‐cell transcriptomics to identify various cell subpopulations in AMI, elucidated the crucial role of macrophages, and combined WGCNA and other analytical methods to identify key genes associated with macrophages as potential biomarkers. Further molecular docking simulations revealed potential therapeutic drugs for AMI, providing guidance for the development of novel treatment strategies for AMI.

**Nomenclature**
AMI: acute myocardial infarction
GEO: Gene Expression Omnibus
WGCNA: weighted gene coexpression network analysis
hdWGCNA: high‐dimensional weighted gene coexpression network analysis
PCA: principal component analysis
ROC: receiver operating characteristic curve
AUC: area under the curve
GSEA: gene set enrichment analysis
TF: transcription factor

## Author Contributions

All authors contributed to this present work. Zhenfang Liu designed the study. Jia Song and Lingling Li acquired the data. Li Yang interpreted the data. Ziyin Fan drafted the manuscript. Genyuan Xie revised the manuscript.

## Funding

The study was supported by Hunan Provincial Natural Science Foundation of China (2023JJ50220).

## Disclosure

All authors read and approved the manuscript.

## Ethics Statement

Ethical approval was not required for this study because it involved no animal or human experiments.

## Consent

The authors have nothing to report.

## Conflicts of Interest

The authors declare no conflicts of interest.

## Data Availability

The datasets generated and/or analyzed during the current study are available in the [GSE66360] repository [https://www.ncbi.nlm.nih.gov/geo/query/acc.cgi?acc=GSE66360] and [GSE270788] repository [https://www.ncbi.nlm.nih.gov/geo/query/acc.cgi?acc=GSE270788].

## References

[1] Frampton J. , Ortengren A. R. , and Zeitler E. P. , Arrhythmias After Acute Myocardial Infarction, Yale Journal of Biology and Medicine. (2023) 96, no. 1, 83–94, 10.59249/LSWK8578, 37009192. 37009192 PMC10052595

[2] Zhang J. , Qian J. , Zhang W. , and Chen X. , The Pathophysiological Role of Receptor-Interacting Protein Kinase 3 in Cardiovascular Disease, Biomedicine & Pharmacotherapy. (2023) 165, 114696, 10.1016/j.biopha.2023.114696, 37329707. 37329707

[3] Parwani P. , Kang N. , Safaeipour M. , Mamas M. A. , Wei J. , Gulati M. , Naidu S. S. , and Merz N. B. , Contemporary Diagnosis and Management of Patients With MINOCA, Current Cardiology Reports. (2023) 25, no. 6, 561–570, 10.1007/s11886-023-01874-x, 37067753. 37067753 PMC10188585

[4] DeFilippis A. P. , Chapman A. R. , Mills N. L. , de Lemos J. A. , Arbab-Zadeh A. , Newby L. K. , and Morrow D. A. , Assessment and Treatment of Patients With Type 2 Myocardial Infarction and Acute Nonischemic Myocardial Injury, Circulation. (2019) 140, no. 20, 1661–1678, 10.1161/CIRCULATIONAHA.119.040631, 31416350. 31416350 PMC6855329

[5] Xia B. , Li Q. , Wu J. , Yuan X. , Wang F. , Lu X. , Huang C. , Zheng K. , Yang R. , Yin L. , Liu K. , and You Q. , Sinomenine Confers Protection Against Myocardial Ischemia Reperfusion Injury by Preventing Oxidative Stress, Cellular Apoptosis, and Inflammation, Frontiers in Pharmacology. (2022) 13, 922484, 10.3389/fphar.2022.922484, 35837272. 35837272 PMC9274168

[6] Krittanawong C. , Khawaja M. , Tamis-Holland J. E. , Girotra S. , and Rao S. V. , Acute Myocardial Infarction: Etiologies and Mimickers in Young Patients, Journal of the American Heart Association. (2023) 12, no. 18, e029971, 10.1161/JAHA.123.029971, 37724944. 37724944 PMC10547302

[7] Zhao W. J. , Qian Y. , Zhang Y. F. , Yang A. H. , Cao J. X. , Qian H. Y. , Liu Y. , and Zhu W. Z. , Endothelial FOSL1 Drives Angiotensin II-Induced Myocardial Injury via AT1R-Upregulated MYH9, Acta Pharmacologica Sinica. (2025) 46, no. 4, 922–939, 10.1038/s41401-024-01410-9, 39592734. 39592734 PMC11950184

[8] Jia D. , Chen S. , Bai P. , Luo C. , Liu J. , Sun A. , and Ge J. , Cardiac Resident Macrophage-Derived Legumain Improves Cardiac Repair by Promoting Clearance and Degradation of Apoptotic Cardiomyocytes After Myocardial Infarction, Circulation. (2022) 145, no. 20, 1542–1556, 10.1161/CIRCULATIONAHA.121.057549, 35430895. 35430895

[9] Epelman S. , Lavine K. J. , Beaudin A. E. , Sojka D. K. , Carrero J. A. , Calderon B. , Brija T. , Gautier E. L. , Ivanov S. , Satpathy A. T. , Schilling J. D. , Schwendener R. , Sergin I. , Razani B. , Forsberg E. C. , Yokoyama W. M. , Unanue E. R. , Colonna M. , Randolph G. J. , and Mann D. L. , Embryonic and Adult-Derived Resident Cardiac Macrophages Are Maintained Through Distinct Mechanisms at Steady State and During Inflammation, Immunity. (2014) 40, no. 1, 91–104, 10.1016/j.immuni.2013.11.019, 2-s2.0-84892450644, 24439267. 24439267 PMC3923301

[10] Frantz S. and Nahrendorf M. , Cardiac Macrophages and Their Role in Ischaemic Heart Disease, Cardiovascular Research. (2014) 102, no. 2, 240–248, 10.1093/cvr/cvu025, 2-s2.0-84899090049, 24501331. 24501331 PMC3989449

[11] Bajpai G. , Bredemeyer A. , Li W. , Zaitsev K. , Koenig A. L. , Lokshina I. , Mohan J. , Ivey B. , Hsiao H. M. , Weinheimer C. , Kovacs A. , Epelman S. , Artyomov M. , Kreisel D. , and Lavine K. J. , Tissue Resident CCR2- and CCR2+ Cardiac Macrophages Differentially Orchestrate Monocyte Recruitment and Fate Specification Following Myocardial Injury, Circulation Research. (2019) 124, no. 2, 263–278, 10.1161/CIRCRESAHA.118.314028, 2-s2.0-85060148825, 30582448. 30582448 PMC6626616

[12] Peet C. , Ivetic A. , Bromage D. I. , and Shah A. M. , Cardiac Monocytes and Macrophages After Myocardial Infarction, Cardiovascular Research. (2020) 116, no. 6, 1101–1112, 10.1093/cvr/cvz336, 31841135. 31841135 PMC7177720

[13] Mills C. D. , Kincaid K. , Alt J. M. , Heilman M. J. , and Hill A. M. , Pillars Article: M-1/M-2 Macrophages and the Th1/Th2 Paradigm, Journal of Immunology. (2017) 199, no. 7, 2194–2201, 10.1093/jimmunol/199.7.2194, 28923981. 28923981

[14] Sica A. , Erreni M. , Allavena P. , and Porta C. , Macrophage Polarization in Pathology, Cellular and Molecular. (2015) 72, no. 21, 4111–4126, 10.1007/s00018-015-1995-y, 2-s2.0-84944170532, 26210152. PMC11113543 26210152

[15] Stuart T. , Butler A. , Hoffman P. , Hafemeister C. , Papalexi E. , Mauck W. M. , Hao Y. , Stoeckius M. , Smibert P. , and Satija R. , Comprehensive Integration of Single-Cell Data, Cell. (2019) 177, no. 7, 1888–1902.e21, 10.1016/j.cell.2019.05.031, 2-s2.0-85066448459, 31178118. 31178118 PMC6687398

[16] Jin S. , Guerrero-Juarez C. F. , Zhang L. , Chang I. , Ramos R. , Kuan C.-H. , Myung P. , Plikus M. V. , and Nie Q. , Inference and Analysis of Cell-Cell Communication Using CellChat, Nature Communications. (2021) 12, no. 1, 10.1038/s41467-021-21246-9, 33597522. PMC7889871 33597522

[17] Yu G. , Wang L. G. , Han Y. , and He Q. Y. , clusterProfiler: An R Package for Comparing Biological Themes Among Gene Clusters, OMICS: A Journal of Integrative Biology. (2012) 16, no. 5, 284–287, 10.1089/omi.2011.0118, 2-s2.0-84860718683, 22455463. 22455463 PMC3339379

[18] Fang F. , Tai R. , Yang F. , Dong R. , and Zhang Y. , Bioinformatic Methods Uncover 5 Diagnostic Biomarkers Associated With Drug Resistance and Metastasis for Gastrointestinal Stromal Tumor, Current Pharmaceutical Analysis. (2025) 21, no. 2, 67–76, 10.1016/j.cpan.2025.01.003.

[19] Aibar S. , González-Blas C. B. , Moerman T. , Huynh-Thu V. A. , Imrichova H. , Hulselmans G. , Rambow F. , Marine J. C. , Geurts P. , Aerts J. , van den Oord J. , Atak Z. K. , Wouters J. , and Aerts S. , SCENIC: Single-Cell Regulatory Network Inference and Clustering, Nature Methods. (2017) 14, no. 11, 1083–1086, 10.1038/nmeth.4463, 2-s2.0-85032583384, 28991892. 28991892 PMC5937676

[20] Langfelder P. and Horvath S. , WGCNA: An R Package for Weighted Correlation Network Analysis, BMC Bioinformatics. (2008) 9, no. 1, 10.1186/1471-2105-9-559, 2-s2.0-60549111634, 19114008. PMC2631488 19114008

[21] Seeliger D. and de Groot B. L. , Ligand Docking and Binding Site Analysis With PyMOL and Autodock/Vina, Journal of Computer-Aided Molecular Design. (2010) 24, no. 5, 417–422, 10.1007/s10822-010-9352-6, 2-s2.0-77953325845, 20401516. 20401516 PMC2881210

[22] Singh A. and Chen R. , The Duration of Oxygen and Glucose Deprivation (OGD) Determines the Effects of Subsequent Reperfusion on Rat Pheochromocytoma (PC12) Cells and Primary Cortical Neurons, International Journal of Molecular Sciences. (2023) 24, no. 8, 10.3390/ijms24087106. PMC10138834 37108268

[23] Singh G. , Siddiqui M. A. , Khanna V. K. , Kashyap M. P. , Yadav S. , Gupta Y. K. , Pant K. K. , and Pant A. B. , Oxygen Glucose Deprivation Model of Cerebral Stroke in PC-12 Cells: Glucose as a Limiting Factor, Toxicology Mechanisms and Methods. (2009) 19, no. 2, 154–160, 10.1080/15376510802355216, 2-s2.0-61649087664, 19778261. 19778261

[24] Ong S. B. , Hernandez-Resendiz S. , Crespo-Avilan G. E. , Mukhametshina R. T. , Kwek X. Y. , Cabrera-Fuentes H. A. , and Hausenloy D. J. , Inflammation Following Acute Myocardial Infarction: Multiple Players, Dynamic Roles, and Novel Therapeutic Opportunities, Pharmacology & Therapeutics. (2018) 186, 73–87, 10.1016/j.pharmthera.2018.01.001, 2-s2.0-85041589954, 29330085. 29330085 PMC5981007

[25] Ning Y. , Huang P. , Chen G. , Xiong Y. , Gong Z. , Wu C. , Xu J. , Jiang W. , Li X. , Tang R. , Zhang L. , Hu M. , Xu J. , Xu J. , Qian H. , Jin C. , and Yang Y. , Atorvastatin-Pretreated Mesenchymal Stem Cell-Derived Extracellular Vesicles Promote Cardiac Repair After Myocardial Infarction via Shifting Macrophage Polarization by Targeting microRNA-139-3p/Stat1 Pathway, BMC Medicine. (2023) 21, no. 1, 10.1186/s12916-023-02778-x, 36927608. PMC10022054 36927608

[26] Song Z. , Yu J. , Wang M. , Shen W. , Wang C. , Lu T. , Shan G. , Dong G. , Wang Y. , and Zhao J. , CHDTEPDB: Transcriptome Expression Profile Database and Interactive Analysis Platform for Congenital Heart Disease, Congenital Heart Disease. (2023) 18, no. 6, 693–701, 10.32604/chd.2024.048081.

[27] Li L. , Cao J. , Li S. , Cui T. , Ni J. , Zhang H. , Zhu Y. , Mao J. , Gao X. , Midgley A. C. , Zhu M. , and Fan G. , M2 Macrophage-Derived sEV Regulate Pro-Inflammatory CCR2^+^ Macrophage Subpopulations to Favor Post-AMI Cardiac Repair, Advanced Science. (2023) 10, no. 14, 2202964, 10.1002/advs.202202964, 36950739. 36950739 PMC10190454

[28] Zheng Y. , Wang Y. , Zou C. , Hu B. , Zhao M. , and Wu X. , Tumor-Associated Macrophages Facilitate the Proliferation and Migration of Cervical Cancer Cells, Oncologie (Tech Science Press). (2022) 24, no. 1, 147–161, 10.32604/oncologie.2022.019236.

[29] Wang D. , Chen H. , and Hu Y. , Polarized Autologous Macrophages (PAM) Can Be a Tumor Vaccine, Oncologie (Tech Science Press). (2022) 24, no. 3, 441–449, 10.32604/oncologie.2022.024898.

[30] Mo Z. , Liu D. , Chen Y. , Luo J. , Li W. , Liu J. , Yu L. , Huang B. , and Zhang S. , Single-cell transcriptomics reveals the role of Macrophage-Naïve CD4 + T cell interaction in the immunosuppressive microenvironment of primary liver carcinoma, Journal of Translational Medicine. (2022) 20, no. 1, 10.1186/s12967-022-03675-2, 36221095. PMC9552358 36221095

[31] Zhang S. , Zhang M. , Goldstein S. , Li Y. , Ge J. , He B. , and Ruiz G. , The Effect of C-Fos on Acute Myocardial Infarction and the Significance of Metoprolol Intervention in a Rat Model, Cell Biochemistry and Biophysics. (2013) 65, no. 2, 249–255, 10.1007/s12013-012-9428-0, 2-s2.0-84874277320, 23054911. 23054911

[32] Huang C. K. , Dai D. , Xie H. , Zhu Z. , Hu J. , Su M. , Liu M. , Lu L. , Shen W. , Ning G. , Wang J. , Zhang R. , and Yan X. , Lgr4 Governs a Pro-Inflammatory Program in Macrophages to Antagonize Post-Infarction Cardiac Repair, Circulation Research. (2020) 127, no. 8, 953–973, 10.1161/CIRCRESAHA.119.315807, 32600176. 32600176

[33] Poggi M. , Canault M. , Favier M. , Turro E. , Saultier P. , Ghalloussi D. , Baccini V. , Vidal L. , Mezzapesa A. , Chelghoum N. , Mohand-Oumoussa B. , Falaise C. , Favier R. , Ouwehand W. H. , Fiore M. , Peiretti F. , Morange P. E. , Saut N. , Bernot D. , Greinacher A. , BioResource N. , Nurden A. T. , Nurden P. , Freson K. , Tregouet D. A. , Raslova H. , and Alessi M. C. , Germline Variants in ETV6 Underlie Reduced Platelet Formation, Platelet Dysfunction and Increased Levels of Circulating CD34+ Progenitors, Haematologica. (2017) 102, no. 2, 282–294, 10.3324/haematol.2016.147694, 2-s2.0-85011589641, 27663637. 27663637 PMC5286936

[34] Xiong X. , Yan Z. , Jiang W. , and Jiang X. , ETS Variant Transcription Factor 6 Enhances Oxidized Low-Density Lipoprotein-Induced Inflammatory Response in Atherosclerotic Macrophages via Activating NF-*κ*B Signaling, International Journal of Immunopathology and Pharmacology. (2022) 36, 10.1177/20587384221076472, 35306921. PMC8943319 35306921

[35] Xu Q. , Liu X. , Wang X. , Hua Y. , Wang X. , Chen J. , Li J. , Wang Y. , Stoeger T. , Chen S. , and Huang N. , Growth Arrest-Specific Protein 7 Regulates the Murine M1 Alveolar Macrophage Polarization, Immunologic Research. (2017) 65, no. 5, 1065–1073, 10.1007/s12026-017-8948-5, 2-s2.0-85029051938, 28895026. 28895026

[36] Ao-Di F. , Han-Qing L. , Xi-Zheng W. , Ke Y. , Hong-Xin G. , Hai-Xia Z. , Guan-Wei F. , and Li L. , Advances in Macrophage Metabolic Reprogramming in Myocardial Ischemia-Reperfusion, Cellular Signalling. (2024) 123, 111370, 10.1016/j.cellsig.2024.111370, 39216681. 39216681

[37] Gladka M. M. , Kohela A. , Molenaar B. , Versteeg D. , Kooijman L. , Monshouwer-Kloots J. , Kremer V. , Vos H. R. , Huibers M. M. H. , Haigh J. J. , Huylebroeck D. , Boon R. A. , Giacca M. , and van Rooij E. , Cardiomyocytes Stimulate Angiogenesis After Ischemic Injury in a ZEB2-Dependent Manner, Nature Communications. (2021) 12, no. 1, 10.1038/s41467-020-20361-3, 33398012. PMC7782784 33398012

[38] Wang W. , Chang S. , He X. , Zhou X. , Shang P. , Chen Y. , Wang X. , Chen L. , Zhang Q. , Qiao Y. , and Feng F. , Sulforaphane Inhibits the Migration and Invasion of BPDE-Induced Lung Adenocarcinoma Cells by Regulating NLRP12, Toxicology and Applied Pharmacology. (2024) 485, 116916, 10.1016/j.taap.2024.116916, 38537874. 38537874

[39] Chen J. G. , Zhu Y. R. , Qian G. S. , Wang J. B. , Lu J. H. , Kensler T. W. , Jacobson L. P. , Muñoz A. , and Groopman J. D. , Fifty Years of Aflatoxin Research in Qidong, China: A Celebration of Team Science to Improve Public Health, Toxins. (2025) 17, no. 2, 10.3390/toxins17020079, 39998096. PMC11860843 39998096

[40] Wang Y. , Petrikova E. , Gross W. , Sticht C. , Gretz N. , Herr I. , and Karakhanova S. , Sulforaphane Promotes Dendritic Cell Stimulatory Capacity Through Modulation of Regulatory Molecules, JAK/STAT3- and MicroRNA-Signaling, Frontiers in Immunology. (2020) 11, 589818, 10.3389/fimmu.2020.589818, 33193420. 33193420 PMC7661638

[41] Wang X. , Chen X. , Zhou W. , Men H. , Bao T. , Sun Y. , Wang Q. , Tan Y. , Keller B. B. , Tong Q. , Zheng Y. , and Cai L. , Ferroptosis Is Essential for Diabetic Cardiomyopathy and Is Prevented by Sulforaphane via AMPK/NRF2 Pathways, Acta Pharmaceutica Sinica B. (2022) 12, no. 2, 708–722, 10.1016/j.apsb.2021.10.005, 35256941. 35256941 PMC8897044

[42] Papini G. , Furini G. , Matteucci M. , Biemmi V. , Casieri V. , Di Lascio N. , Milano G. , Chincoli L. R. , Faita F. , Barile L. , and Lionetti V. , Cardiomyocyte-Targeting Exosomes From Sulforaphane-Treated Fibroblasts Affords Cardioprotection in Infarcted Rats, Journal of Translational Medicine. (2023) 21, no. 1, 10.1186/s12967-023-04155-x, 37161563. PMC10169450 37161563

